# Population genetic structure of the Mediterranean horseshoe bat *Rhinolophus euryale* in the central Balkans

**DOI:** 10.1371/journal.pone.0210321

**Published:** 2019-01-30

**Authors:** Ivana Budinski, Jelena Blagojević, Vladimir M. Jovanović, Branka Pejić, Tanja Adnađević, Milan Paunović, Mladen Vujošević

**Affiliations:** 1 Department of genetic research, Institute for biological research “Siniša Stanković”, University of Belgrade, Belgrade, Serbia; 2 Department of biological collections, Natural History Museum, Belgrade, Serbia; University of Reunion Island, RÉUNION

## Abstract

Migratory behaviour, sociality and roost selection have a great impact on the population structure of one species. Many bat species live in groups, and movements between summer and hibernation sites are common in temperate bats. The Mediterranean horseshoe bat *Rhinolophus euryale* is a cave-dwelling species that exhibits roost philopatry and undertakes seasonal movements which are usually shorter than 50 km. Its distribution in Serbia is restricted to karstic areas in western and eastern parts of the country, with a lack of known roosts between them. In this study, microsatellite markers were used to evaluate genetic variation in this species in the Central Balkans. Specifically, spatial genetic structuring between geographic regions and relatedness within different colony types were assessed. All analysed loci were polymorphic, and there was no significant inbreeding coefficient recorded. A moderate degree of genetic differentiation among the sampled colonies was found, and significant isolation by distance was recorded. Our results revealed that populations show a tendency to segregate into three clusters. Unexpectedly, populations from Montenegro and Eastern Serbia tended to group into one cluster, while populations from Western Serbia and Slovenia represented second and third cluster, respectively. The majority of variance was partitioned within colonies, and only a small but significant portion among clusters. Average relatedness within colony members was close to zero, did not differ significantly between the different colony types, and kinship is unlikely to be a major grouping mechanism in this species.

## Introduction

Bats represent one of the most peculiar groups of mammals due to specific features such as nocturnality, self-powered flight and echolocation [1]. Numerous molecular genetic tools developed in previous years have been successfully used to address various questions concerning evolution, ecology and behaviour of this group of mammals [2]. Different factors like seasonal movements, roosting biology, mating strategies and dispersal patterns shape the genetic structure of each species [3]. Comparing the genetic characteristics of individuals from various sites can provide information on population structuring and the degree of gene flow within and across them [4]. Knowledge on species’ genetic structure is a key information to infer future dynamics of the species, and to generate appropriate recommendations for conservation.

The vast majority of bat species live in mixed-sex groups, at least for some part of their annual breeding cycle, and in temperate species females commonly aggregate in summer maternity colonies for communal breeding [3,5,6]. During that period males are usually solitary, but in some species they may form groups [7]. Sexual segregation during the summer might be due to different thermal requirements or avoidance of food competition between males and females [8]. Several factors have been proposed as potential drivers of grouping in bats, such as resource limitation and defence from predators [6,9], information transfer [10], social thermoregulation [11] and kinship [12]. Roost philopatry has been recorded in many bat species, and usually females are the sex that shows fidelity to their natal roosts [3]. Female philopatry has been confirmed in some European bat species: *Myotis bechteinii* [12], *M*. *myotis* [13], *M*. *nattereri* [14], *Plecotus auritus* [15], *Rhinolophus ferrumequinum* [16]. If females exhibit natal philopatry, and formation of a maternal colony is promoted by kin-biased behaviour, it is expected that relatedness among individuals within groups would be higher than for random associations of animals [5,6].

Moussy *et al*. [17] defined migration as “a regular two-way movement of populations between regions, one of which usually includes the breeding site”. In temperate bat species seasonal migration occurs between hibernation and summer nursery roosts [3]. Some bat species move”locally” and are considered to be sedentary, while others exhibit migratory behaviour and fly over long distances [18]. Migratory behaviour can have a great effect on population structure. Migratory species usually show weak genetic structuring (0–5% of differentiation between pairs of distant populations), suggesting high gene flow across their distribution [3]. In contrast, populations of non-migratory species typically exhibit high levels of genetic structuring due to restricted gene flow among distinct populations which may lead to isolation by distance (reviewed in Burland & Worthington Wilmer [3] and Moussy *et al*. [17]).

*Rhinolophus euryale* is a cave-dwelling medium-sized horseshoe bat present in the Mediterranean area, with distribution mostly limited to karstic regions [19]. It is considered to be a sedentary species, undertaking seasonal movements up to 50 km [18], with exceptionally long movements recorded in Italy (83 km [20]) and in France (134 km [21]). Similar to other *Rhinolophus* species, it has wing morphology that limits long-range movements [3,22]. Females form nursery colonies during summer, while in winter both sexes aggregate in colonies for hibernation [19]. Roost fidelity has been recorded in both sexes ([23], personal observation).

The distribution of *R*. *euryale* in Serbia overlaps with karstic areas [24], that extend in western (Dinarides) and eastern (Carpatho-Balkanides) Serbia [25], with a lack of underground roosts, especially caves, in central parts of the country. All but one recently known roosts are in caves ([24], personal observation). Sedentary nature of this species, low vagility and the existence of roost philopatry might lead to restricted gene flow between populations that are geographically more distant. In addition to geographic distance, lack of suitable roosts in central Serbia could be a limiting factor for movements between Western and Eastern Serbia. Therefore, we assume low levels of gene flow between these regions and more intensive gene flow within them. Apart from hibernation and nursery roosts, two male summer colonies of this species have been observed in Serbia. One is in Eastern Serbia (Bela Sala) and has existed for at least 20 years (it was discovered in 1996). It comprises up to 100 animals (sometimes mixed with *R*. *blasii*; personal observation) and was previously reported by Uhrin *et al*. [23]. Another one was discovered in Western Serbia (Tmuša) in 2013 and has been monitored since. Its size varies between 100–300 animals. All specimens captured from those roosts during summer were exclusively males.

To the best of our knowledge, there is only one published study concerning genetic analysis of the Mediterranean horseshoe bat [26] that discussed its phylogeography in Southeastern Europe and Anatolia. There are no other available published data on the genetic structure of this species. The goals of our study were: (i) to assess genetic variation in populations of the Mediterranean horseshoe bat in the Central Balkans using microsatellite markers; (ii) to analyse levels of genetic differentiation between bats from Western and Eastern Serbia and to test predictions that populations of this species exhibit spatial genetic structuring and isolation by distance (iii) to evaluate relatedness among colony members within different colony types (nursery, male summer and hibernation) and to examine whether kinship is driving group formation.

## Materials and methods

### Ethics statement

*Rhinolophus euryale* is a protected species in Serbia, Montenegro and Slovenia, and capturing and sampling were carried out under the licences provided by the responsible authorities in Serbia, Montenegro and Slovenia (list of licences given in S1 File). According to Serbian, Montenegrian and Slovenian laws, no further ethical approval by a committee is required for aforementioned procedures, which were carried out in accordance with the species-specific recommendations of the Canadian Council on Animal Care [27]. All bats were successfully released in good condition at the capture site immediately after processing.

### Sample collection

**Fig 1:**
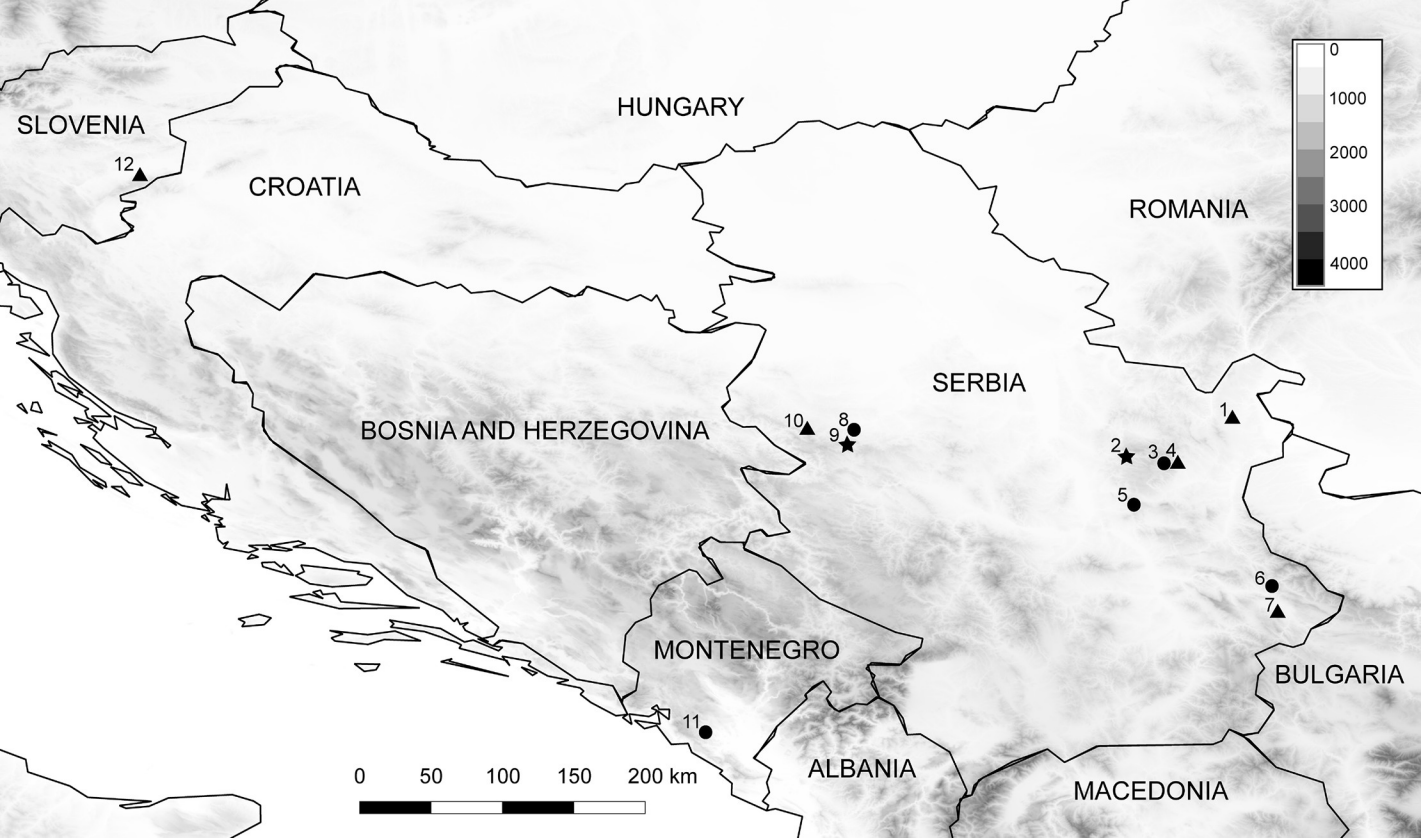
Map of sampled localities. Locality numbers correspond to names in Table 1. triangle–hibernation roost, star–male summer roost, circle–nursery roost. Elevation in meters corresponding to the greyscale is given in legend.

During the period 2012–2016, a total of 254 bat samples were collected from twelve localities (Table 1, Fig 1), with distances from each other ranging between 6.4 and 424 km. Ten sites were sampled in Serbia, and two additional ones in Montenegro and Slovenia. All but one of the roosts sampled were caves. Bats were captured during emergence using mist-nets set up at the roost entrance and/or using a hand net inside the roost. All specimens were identified to species level, sexed and classified as adults or juveniles according to the degree of ossification of the epiphyseal plates on finger bones [28]. Reproductive conditions of adults were assessed following Racey [29]. Forearm length and body mass of the captured animals were measured to the nearest 0.05 mm and 0.25 g, respectively.

**Table 1:** List of sampled localities in the geographic regions with number of specimens sampled and colony type.

Locality no.	Locality name	Geographic region	No. of specimens	Colony type	Sampling date
1	Canetova pećina	ES	19	Hibernation	24.4.2013.
2	Bela Sala	ES	21	Male summer	18.6.2012.
3	Ljubinkova pećina	ES	35	Nursery	3.8.2013.
4	Lazareva pećina	ES	24	Hibernation	8.3.2013.
5	Pećurski kamen	ES	18	Nursery	5.9.2015.
6	Temska	ES	24	Nursery	13.5.2013.
7	Držinska pećina	ES	40	Hibernation/transitory	15.5.2013.
8	Petnička pećina	WS	23	Nursery	13.6.2013. 5.7.2013.
9	Tmuša	WS	15	Male summer	13.7.2015.
10	Drenajićka pećina	WS	10	Hibernation	18.9.2014.
11	Začirska pećina	MNE	12	Hibernation/transitory	14.9.2013.
12	Kostanjeviška jama	SLO	13	Hibernation	16.4.2016.

ES–Eastern Serbia, WS–Western Serbia, MNE–Montenegro, SLO–Slovenia

Tissue samples were taken from each plagiopatagium using 3-mm biopsy punches following Worthington Wilmer & Barratt [30] and stored in 99% ethanol.

### Microsatellite analysis

Genomic DNA was extracted using Quick-gDNA Mini Prep Kit (Zymo-Research) according to the manufacturer’s protocol, following overnight digestion with proteinase K [31]. All individuals were genotyped for eight nuclear microsatellite loci: RE007, RE017, RM002, RM003, RM010, RM011, RM015 and RM025 [32]. Six loci (all but RM002 and RM010) were amplified together in a 10 μl multiplex PCR containing 1 μl of DNA template, 1 x Multiplex PCR Master Mix (QIAGEN) and the primer concentrations given in Puechmaille *et al*. [32], following the amplification conditions described in Puechmaille *et al*. [33]. Two loci (RM002 and RM010) were amplified in separate 10 μl reactions containing 1 μl of DNA template, 1 x GoTaq Reaction Buffer (Promega), 1.5 mM Mg^2+^, primers RM002 and RM010 in concentrations as in Puechmaille *et al*. [32], 0.5 mM dNTPs and 0.5 U of GoTaq DNA Polymerase (Promega), with the following reaction conditions: 3 min at 95°C, 40 cycles at 95°C for 30 s, 60 ^o^C for 30 s, 72 ^o^C for 30 s and a final elongation at 72 ^o^C for 10 min. PCR products were stored at 4 ^o^C until genotyping. The PCR products were run on a four-capillary ABI 3130 Genetic Analyser (Applied Biosystems) with 500-LIZ size standard (Applied Biosystems), and genotypes were obtained using the GeneMapper 4.0 (Applied Biosystems). The microsatellite data was submitted to the Figshare and assigned a DOI: https://doi.org/10.6084/m9.figshare.7093646.

Bayesian clustering procedure implemented in STRUCTURE 2.3.4. [34] was carried out to assess overall population structuring (i.e. infer the number of genetic clusters in the entire dataset). Analyses were run assuming the admixture ancestry model using sampling locations as prior, and correlated allele frequencies with default settings. To obtain the optimal number of clusters (K), ten MCMC runs for each K (K = 1–12) were performed, the length of burn-in was 100000 followed by 1000000 iterations. The probability of each K was determined using Evanno’s ΔK statistics [35] implemented in STRUCTURE HARVESTER 0.6.94 [35]. Following Puechmaille [36] recommendations for uneven sampling among sites, subsampling of genotypes was performed before running the analyses. Fifteen individuals per sampling site were randomly chosen without replacement, or all individuals per site were analysed if the sample size was ≤15. Moreover, additional parameters MaxMeaK and MaxMedK [36] were calculated to determine true number of clusters using value 0.5 as threshold of mean membership coefficient. R package GENELAND 4.0.7. [37] was used to test for spatial population structure taking into account information on the geographic location of individual samples. Ten simultaneous independent MCMC runs were executed with 1000000 iterations, thinned every 10000^th^ iteration and postprocessed with 10% burn-in. The number of populations was set 1–12 with correlated allele frequencies and true spatial and null models. The optimal K was selected based on highest posterior mean density across runs.

Analysis of genetic diversity, including the number of alleles per locus (N_A_), allelic richness (R), observed (H_O_) and expected (H_E_) heterozygosities and inbreeding coefficient (F_IS_) were calculated in GenAlEx 6.503 [38] and FSTAT 2.9.3.2 [39]. Departures from Hardy–Weinberg equilibrium (HWE) and assessment of linkage disequilibrium were tested in GENEPOP 4.6.9 [40]. Micro-checker 2.2.3 [41] was used to test for errors and null alleles, and global and pairwise F_ST_ values corrected for null alleles were calculated using FreeNA software [42]. Both uncorrected and null-allele corrected data were analysed and, since the results did not differ significantly, only the uncorrected data are shown herein. Genetic differentiation among pairs of populations was measured with Weir & Cockerham [43] pairwise F_ST_ estimation in Arlequin 3.5.2.2 [44], treating each colony as a distinct subpopulation. The maximum value of F_ST_ may not reach 1 when examining highly polymorphic loci [45]. To assess more accurate estimation of population substructuring, Jost's D [45] and G''_ST_ [46] were calculated in GenAlEx 6.503 [38] based on 999 permutations. The same software was used to calculate pairwise Jost's D and G''_ST_ among clusters obtained in STRUCTURE analysis. Analysis of molecular variance (AMOVA) was performed in Arlequin 3.5.2.2 [44] to test for genetic differentiation among genetic clusters revealed by clustering analyses. Isolation by distance was tested using the Mantel test implemented in the same software. Geographic distances were estimated as the Euclidean distances between localities, and genetic distances were linearized F_ST_ as F_ST_/(1-F_ST_). The same matrix of geographic distances was used for additional Mantel tests that were performed in the zt 1.1 software [47] with 10000 randomized permutations, using Jost's D and G''_ST_ values as genetic distance matrices. Moreover, to account for the effect of both geographic and environmental factors on genetic differentiation, partial Mantel test based on Pearson’s product-moment correlation was performed in vegan 2.5–2 R package [48]. G''_ST_ values and Euclidean distances between localities were used as genetic and geographic distances, respectively. To estimate environmental differences, 19 bioclimatic variables (30-arc seconds (~1 km^2^) layer resolution) for current conditions were obtained from WorldClim2 [49] for each locality. Variables were summarized using PCA, and Euclidean distances were calculated. First PCA component was retained representing 54.81% of the total variation.

Sex-biased dispersal was investigated using *sexbias* function in hierfstat R package [50]. Mean of corrected assignment indices (mAIc), variance in assignment (vAIC), F_IS_ and F_ST_ values were compared between sexes in two-sided test with 10000 permutations. Contemporary migration rates among geographic regions (Table 1) were estimated using a Bayesian approach implemented in BayesAss 3.0.3 [51]. Analysis was run for 30000000 MCMC iterations with 6000000 burn-in, sampling every 1000^th^. Mixing parameters were adjusted to m = 0.2, a = 0.5, f = 0.6 to obtain the recommended acceptance rates. MCMC chain mixing and convergence were determined visualizing the trace file in Tracer 1.7 [52].

Relatedness between individuals within and among colonies was calculated by the pairwise relatedness estimator of Queller & Goodnight [53] in Coancestry 1.0.1.8 [54], while one-way ANOVA was performed in Statistica 5.1 [55] to test for differences in relatedness among animals within different colony types.

## Results

**Fig 2:**
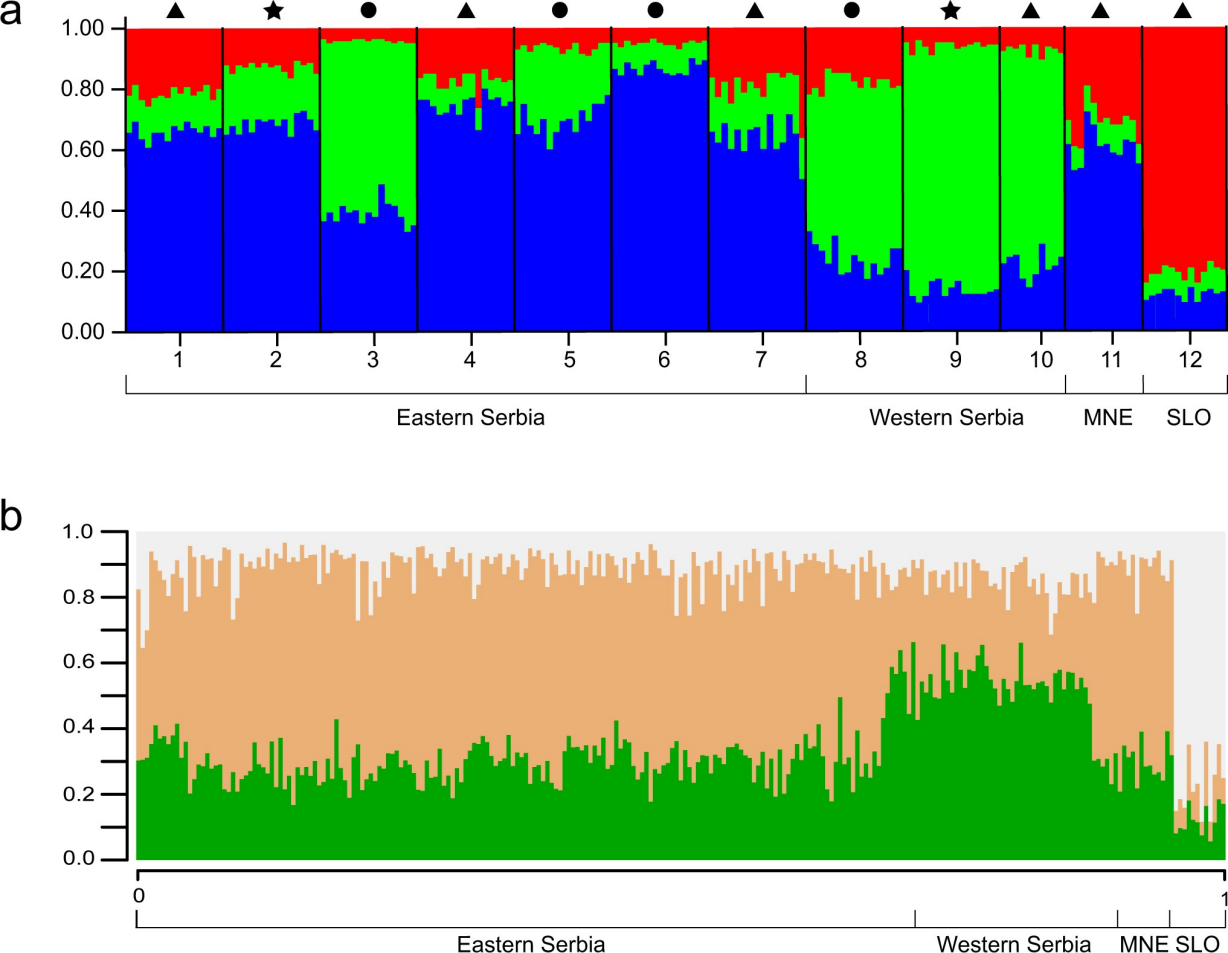
Assignment probability of each individual to a given population. a) STRUCTURE plot under assumptions of K = 3. Population numbers correspond to names in Table 1; triangle–hibernation roost, star–male summer roost, circle–nursery roost. b) GENELAND admixture proportions. Labelled geographic regions are as in Table 1.

Genetic clustering analyses in STRUCTURE following Evanno’s ΔK method indicated the existence of three clusters (Fig 2A, S1 Fig), which was supported by the MaxMeaK and MaxMedK calculations (S2 and S3 Files). Plots representing the assignment probability of each individual to a given population under several assumptions of K are given in S2 Fig. Likewise, when information about geographic location of sampled populations was taken into account, existence of three genetic clusters was revealed. Although a lot of admixture is evident across the sampled area (Fig 2), populations still show a tendency to segregate in slightly different groups. All but one populations from Eastern Serbia clustered together with population from Montenegro, while western Serbian populations grouped with remaining population from Eastern Serbia into second genetic cluster. Population from Slovenia represented the third cluster. This clustering tendency was also obtained on maps of posterior probabilities of cluster memberships in GENELAND (results not shown).

All analysed loci were polymorphic with 7–13 alleles per locus, and observed heterozygosities were in the range 0.482–0.835 (Table 2). Significant departures from Hardy-Weinberg equilibrium were detected for loci RM003 and RM011. F_IS_ was also significantly greater than zero in these loci. This was probably caused by the presence of null alleles, as null alleles were detected in both loci in 50% of the sampled populations. None of the analysed loci were in linkage disequilibrium.

**Table 2:** Summary statistics for eight microsatellite loci.

Locus	N_A_	R	H_O_	H_E_	F_IS_	H-W
RE007	9	5.80	0.794	0.802	-0.006	n.s.
RE017	8	4.07	0.741	0.695	-0.080	n.s.
RM002	13	5.95	0.796	0.804	0.023	n.s.
RM003	11	6.24	0.609	0.819	0.254	***
RM010	12	7.35	0.835	0.865	0.031	n.s.
RM011	8	4.47	0.482	0.685	0.312	***
RM015	7	4.03	0.669	0.687	0.017	n.s.
RM025	8	4.41	0.629	0.631	-0.019	n.s.

N_A_−number of alleles, R–allelic richness, H_O_−observed heterozygosity, H_E_−expected heterozygosity, F_IS_−fixation index, H-W–significance of tests for HWE departures (*** significant at the p < 0.05 level, n.s.–not significant)

At the population level, gene diversity and observed heterozygosity were similar among populations (Table 3). F_IS_ estimates at the colony levels did not differ significantly from zero, indicating that there was no significant inbreeding within these colonies. There were no significant differences in genetic variability (number of alleles, heterozygosity and inbreeding coefficient) among the different colony types.

**Table 3:** Genetic variability within the sampled colonies.

Colony	NA	H_O_	H_E_	F_IS_
1	6.875	0.724	0.734	0.041
2	6.375	0.679	0.716	0.081
3	7.625	0.725	0.749	0.047
4	6.500	0.724	0.745	0.049
5	6.375	0.660	0.717	0.108
6	6.500	0.719	0.737	0.054
7	7.500	0.703	0.738	0.064
8	6.125	0.690	0.728	0.080
9	5.750	0.667	0.704	0.101
10	5.500	0.622	0.685	0.146
11	6.000	0.708	0.727	0.068
12	5.875	0.740	0.737	0.036

N_A_−number of different alleles averaged over loci, H_O_−observed heterozygosity, H_E_−expected heterozygosity, F_IS_−inbreeding coefficient.

Pairwise F_ST_ values among the sampled colonies were low, ranging from -0.004 to 0.064 (Table 4). Pairwise Jost's D and G''_ST_ revealed greater levels of differentitation between pairs of populations. Values for both parameters were similar, and only Jost's D values are presented herein (Table 4), and all pairwise estimates of population differentiation with significance levels are shown in S1–S3 Tables. Slovenian colony, that is the most geographically distant one, differed significantly from all other colonies. The colony from Montenegro differed significantly from colonies in Western Serbia, but not from Eastern Serbian ones. With a single exception, colonies within one geographic region did not differ significantly, and there was no significant population structure associated with different roost type. Additionally, both Jost's D and G''_ST_ revealed significant differentiation among clusters (S4 Table).

**Table 4:** Pairwise F_ST_ and Jost's D distances. F_ST_ values are below, and Jost's D above the diagonal.

	1	2	3	4	5	6	7	8	9	10	11	12
1	-	0.007	0.010	**0.044**	-0.011	-0.005	0.014	**0.102**	**0.119**	**0.126**	-0.039	**0.119**
2	0.004	-	0.001	-0.009	-0.012	-0.011	0.002	**0.074**	**0.045**	0.038	0.028	**0.099**
3	0.004	0.002	-	0.000	0.004	-0.008	0.001	**0.069**	**0.030**	0.047	0.025	**0.088**
4	**0.015**	-0.001	0.001	-	**0.040**	-0.011	0.012	**0.073**	**0.058**	0.057	0.014	**0.083**
5	-0.002	-0.001	0.003	**0.015**	-	0.000	0.003	**0.086**	**0.066**	0.063	0.014	**0.097**
6	0.000	-0.002	-0.002	-0.002	0.002	-	-0.015	**0.070**	**0.079**	**0.073**	-0.011	**0.100**
7	0.005	0.002	0.002	0.005	0.003	-0.004	-	**0.052**	**0.050**	**0.052**	0.028	**0.127**
8	**0.036**	**0.027**	**0.024**	**0.024**	**0.032**	**0.024**	**0.020**	-	-0.005	**0.058**	**0.070**	**0.113**
9	**0.041**	**0.019**	0.012	**0.022**	**0.026**	**0.029**	**0.021**	0.000	-	0.010	**0.086**	**0.137**
10	**0.042**	0.016	0.014	0.019	0.024	**0.023**	0.018	0.020	0.007	-	**0.168**	**0.156**
11	-0.011	0.011	0.010	0.006	0.008	-0.001	0.011	**0.026**	**0.032**	**0.058**	-	**0.113**
12	**0.055**	**0.052**	**0.045**	**0.045**	**0.050**	**0.052**	**0.058**	**0.054**	**0.062**	**0.064**	**0.054**	-

Values in bold indicate differentiations that are significantly greater than expected by random at p < 0.05. Population numbers correspond to ones given in Table 1. Darker shades of grey indicate the greater distance between localities □ 0–150 km, ■ 151–300 km, ■ 301–450 km and ■ > 450 km.

AMOVA suggested that the majority of genetic variation was partitioned within colonies (95.3%, p < 0.001), and only a small portion among colonies within groups (0.32%, p = 0.235) and among them (4.38%, p < 0.001). Examination of isolation by distance suggested significant correlation (Mantel r = 0.732, p < 0.01) between genetic and geographic distances of Mediterranean horseshoe bat colonies. Concordant results were obtained in additional Mantel tests (performed in zt software), and they are not shown herein. On the other hand, the removal of the effect of geographic variation in the partial Mantel test resulted in a non-significant correlation between genetic and environmental distances (r = -0.228, p = 0.871) (S3 Fig).

Our study revealed no significant sex-biased dispersal; results in all four performed tests were statistically insignificant. BayesAss analysis suggests that there has not been significant dispersal between geographic regions within the last two generations.

Mean relatedness within colony members was close to zero in all analysed samples. The proportion of relatives within colony members (r ≥ 0.25) ranged between 8.5–16.21%, 9.52–17.68% and 4.54–20%, within the nursery, summer male and hibernation colonies, respectively. There was no significant effect of colony type on relatedness among colony members [F(2,9) = 1.00, p = 0.405], nor on percentage of relatives within colonies [F(2,9) = 0.13, p = 0.883].

## Discussion

The results of this study revealed polymorphic loci and the absence of significant inbreeding in all analysed populations of the Mediterranean horseshoe bat. However, in spite of its flight ability, *R*. *euryale* displayed genetic substructuring across the analysed range. Isolation by distance was demonstrated in this species, with the greatest genetic differences observed between the samples from Western Serbia and Slovenia. Isolation by distance has also been detected in congeneric species *Rhinolophus ferrumequinum* across the species range [56], and in other sedentary bat species such as *Myotis bechsteinii* [57], *M*. *myotis* [13] and *Plecotus auritus* [58].

A lot of admixture was detected in Central Balkans and there was no obvious pattern of genetic differentiation. However, our analyses revealed existence of three groups (Slovenia, Western Serbia and Eastern Serbia together with Montenegro), and this clustering pattern was supported by various analyses. Although existence of three genetic clusters was statistically highly supported, they were not geographically coherent and not easy to differentiate by looking at the admixture levels. Additionally, asigning populations from Montenegro and Eastern Serbia to the same cluster does not seem intuitive. Franz *et al*. [59] showed that, when data set is characterized by isolation by distance, obtained clusters are sometimes difficult to explain biologically.

Western Serbian group was always first to segregate in cluster analyses and these populations showed significant differentiation from almost all the populations from another two groups. Distances between colonies in Western and Eastern Serbia are greater than 100 km and the habitat between them is devoid of caves, thus it had been assumed that this would represent a barrier to gene flow. Within the Carpathian region, Uhrin *et al*. [23] recorded seasonal movements up to 16.7 km, while Dietz *et al*. [60] found that distances between hibernacula and summer roosts can be larger (58.8 km) in Bulgaria. As previously assumed, lower genetic differentiation was observed within geographic regions than among them. This finding is supported by mark-recapture data from Serbia, where seasonal movements in this species have been recorded only within geographic regions (Eastern and Western Serbia), but not between them ([24], personal observation). Correspondingly, BayesAss results did not reveal significant migration of animals among these regions within the last two generations.

Another interesting finding is that genetic differentiation between samples from Western Serbia and Montenegro (within Dinarides) was significant and far more pronounced than between those from Montenegro and Eastern Serbia (between Dinarides and Carpatho-Balkanides). High mountains in the north of Montenegro might act as a physical barrier, and to a certain extent restrict gene flow between colonies within Dinarides. Maybe the most peculiar discovery is the genetic similarity between populations from Eastern Serbia and Montenegrin population. According to significant isolation by distance that was confirmed in our study, it would be expected that these geographically distant populations (distances among roosts range 270–345 km) would be more genetically different. Furthermore, there was no significant correlation between genetic and environmental differences, and observed genetic pattern could not be explained with environmental factors. It is still unclear why these populations are genetically very similar. They showed a tendency to group together in clustering analyses and there were no significant differentiations between pairs of populations, suggesting that there might be gene flow occurring between them. Gene flow throughout Albania, Kosovo and southern Serbia could be a possible explanation for population connectivity between these regions. *R*. *euryale* has been recorded near Skadar lake and the Korab area in Albania (P. Théou, personal communication), and there is a historical finding of this species in Kosovo, near the town of Peć [24]. Due to lack of more data on its distribution in Albania and Kosovo, the proposed assumption remains to be tested. There are not known roosts of this species in Southern Serbia (non-karstic area). Nonetheless, it is possible that *R*. *euryale* could be using alternative roost types such as roof spaces of buildings (as described for the Carpathian region by Uhrin *et al*. [23]), or abandoned mines or quarries. Alternatively, another explanation for genetic similarities between these two regions could be a potential microsatellite homoplasy. Different copies of a locus that have identical size but are not necessarily identical by descent can mislead estimation of gene flow and differentiation between geographically distant populations [61].

Moderate genetic differentiation among colonies of this species was found, that can be explained by non-migratory behaviour and roost philopatry, together with isolation by distance. Similar to other *Rhinolophus* species, Mediterranean horseshoe bat has a low wing loading and aspect ratio, causing a long-distance flight to be energetically inefficient [3,22]. Roost philopatry has previously been recorded in this species; females show fidelity to maternity roosts, and both sexes to hibernacula ([23,24], personal observation). Furthermore, one case of male fidelity to bachelor roost was recorded (male was recaptured after 12 years, personal observation). Even though sedentary and philopatric species are expected to be more prone to inbreeding depression [62], our findings did not support that prediction. Similar results were found for other sedentary bat species where this question was addressed, and male-biased dispersal and extra-colonial mating have been proposed as mechanisms that prevent inbreeding depression [62,63]. Our results did not show evidence of sex-biased dispersal.

Our results disclosed very low (close to zero) mean colony relatedness values. Evidence of roost fidelity united with low values of relatedness within colony members has already been reported in several other temperate bat species, e.g. *Myotis bechsteinii* [64], *Nyctalus leisleri* [5], *Plecotus auritus* [15], *Rhinolophus ferrumequinum* [16]. Kerth *et al*. [63] proposed that it is a consequence of low male reproductive skew and outbreeding. All aforementioned studies were conducted on maternity colonies. Relatedness studies on swarming bat species [4,14,65] showed that average colony relatedness values were higher in maternity than in swarming colonies, suggesting that high gene-flow occurs at swarming sites. Mating in *R*. *euryale* usually occurs in autumn transient roosts or in hibernacula [19], and under the assumption that animals from different summering sites gather together for mating and hibernation, higher genetic richness should be found in such colonies. Nevertheless, this was not confirmed in our study.

In spite of natal philopatry to nursery roosts among females, our results showed that a large portion of individuals within colonies were non-relatives, and thus kinship is probably not a major factor underlying the formation of female groups. Benefits from group thermoregulation and sharing knowledge about accessible foraging areas are more likely to play important roles in establishing nursery colonies [11,16]. Relatedness among animals within male summer colonies did not differ from those in other colony types and, as with females, kinship is probably not driving male grouping. Few studies addressed questions about which factors underlie male sociality during summer [66]. Safi & Kerth [66] disclosed that male coloniality is coupled with increased foraging success rather than group thermoregulation. However, their results refer to open space foraging species, and *R*. *euryale* is adapted for foraging in cluttered environments [22]. Thus, this might not be applicable to the Mediterranean horseshoe bat, and the question of male sociality in this species still remains open.

Our study represents the first population genetics study of the Mediterranean horseshoe bat using microsatellite markers. The observed genetic diversity was high, but significant isolation by distance and moderate spatial population structuring were found. As previously reported, the most stable and largest populations of this species occur on the Iberian and Balkan peninsulas [22], and protecting Balkan populations might be a vital step for the sustentation of this species. The underlying factors determining gathering into colonies are still unclear, but animal aggregations do not appear to be driven by kinship in this species.

## Supporting information

S1 File List of permits for capturing and sampling given by the responsible authorities. (DOC)

S2 File Calculations of MaxMeaK using value 0.5 as threshold of mean membership coefficient. (XLS)

S3 File Calculations of MaxMedK using value 0.5 as threshold of mean membership coefficient. (XLS)

S1 Fig Plot of Delta K values from STRUCTURE analysis. (TIF)

S2 Fig STRUCTURE plots under assumptions of different K. (TIF)

S3 Fig **Plots of simple and partial Mantel tests showing the relationships between a) geographic and genetic distances, b) environmental and genetic distances.** Triangle–pairs of populations belonging to the same genetic cluster, circle–pairs of populations belonging to differemt genetic clusters (under assumption that populations from Eastern Serbia and Montenegro belong to the same cluster). Red triangles represent pairs of populations within Eastern Serbia, and blue triangles pairs of populations from Eastern Serbia with Montenegrin population.
(TIF)

S1 Table Pairwise F_ST_ values (under diagonal) and p values for pairwise genotypic differentiation (above the diagonal). (DOC)

S2 Table Pairwise Jost's D values (under diagonal) and p values for pairwise genotypic differentiation (above the diagonal). (DOC)

S3 Table Pairwise G''_ST_ values (under diagonal) and p values for pairwise genotypic differentiation (above the diagonal). (DOC)

S4 Table Pairwise G''_ST_ values (under diagonal) and Jost's D values (above diagonal) among 3 genetic clusters revealed by STRUCTURE. (DOC)
