# Supplementary material for: Population genetic structure of the Mediterranean horseshoe bat Rhinolophus euryale in the central Balkans
Source: PLoS One. 2019 Jan 30;14(1):e0210321. doi: 10.1371/journal.pone.0210321 (PMC6353099; doi:10.1371/journal.pone.0210321)
Supplement: S1 File — (DOC) [file pone.0210321.s001.doc]

**SERBIA**

Permit no. 353-01-3031/2012-03, given by the Ministry of Environment, Mining and Spatial Planning of Serbia

Permit no. 353-01-685/2013-08, given by the Ministry of Energetics, Development and Environmental Protection of Serbia

Permit no. 353-01-2660/2013-08, given by the Ministry of Energetics, Development and Environmental Protection of Serbia

Permit no. 353-01-1994/2014-17, given by the Ministry of Agriculture and Environmental Protection of Serbia

Permit no. 353-01-2503/2015-17, given by the Ministry of Agriculture and Environmental Protection of Serbia

MONTENEGRO

Permit no. 02-UPI-959/4, given by the Environmental Protection Agency of Montenegro

SLOVENIA

Permit no. ARSO 35601-35/2010-6, given by the Ministry of the Environment and Spatial Planing of Slovenia
