# Supplementary figures and images for: Population genetic structure of the Mediterranean horseshoe bat Rhinolophus euryale in the central Balkans
Source: PLoS One. 2019 Jan 30;14(1):e0210321. doi: 10.1371/journal.pone.0210321 (PMC6353099; doi:10.1371/journal.pone.0210321)

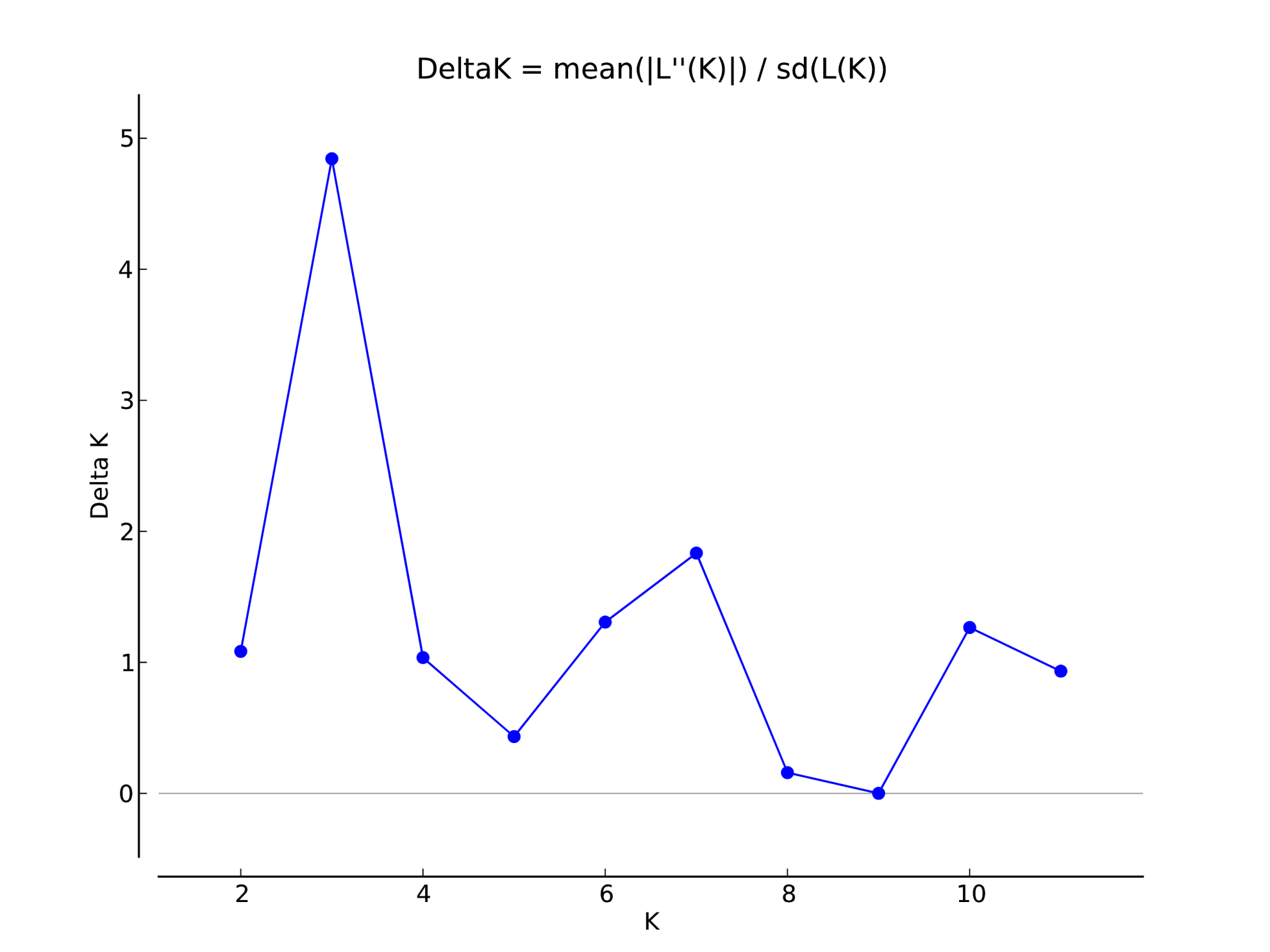

Supplement: S1 Fig — (TIF) [file pone.0210321.s004.tif]

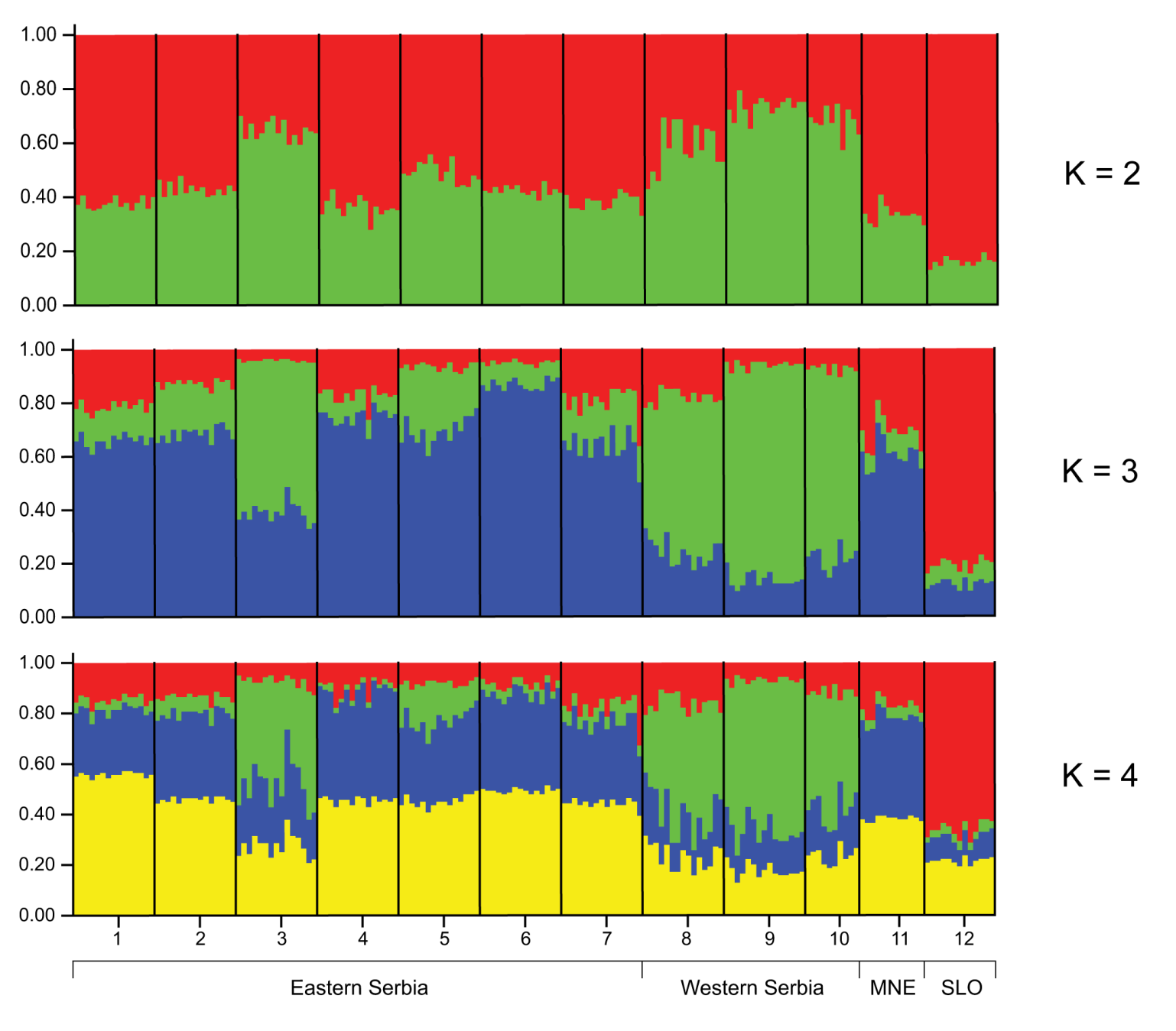

Supplement: S2 Fig — (TIF) [file pone.0210321.s005.tif]

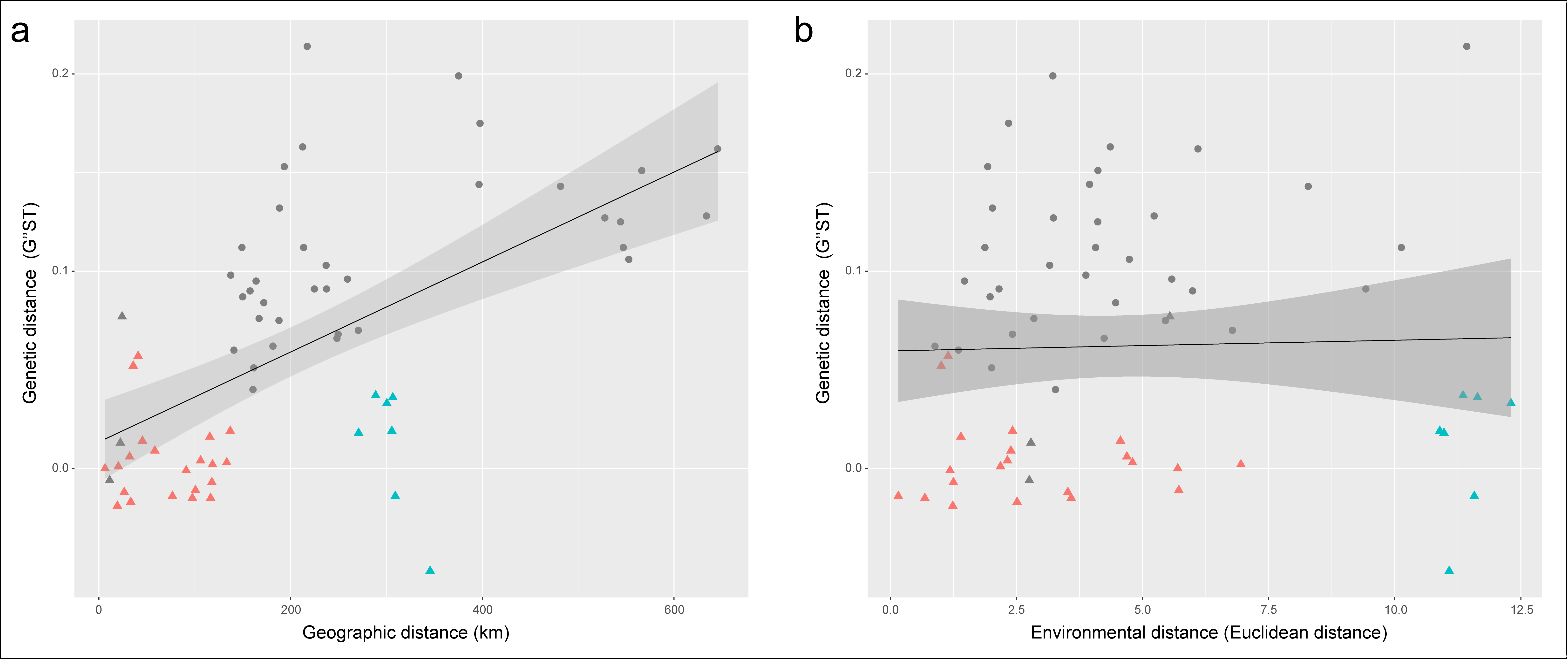

Supplement: S3 Fig — Plots of simple and partial Mantel tests showing the relationships between a) geographic and genetic distances, b) environmental and genetic distances. Triangle–pairs of populations belonging to the same genetic cluster, circle–pairs of populations belonging to differemt genetic clusters (under assumption that populations from Eastern Serbia and Montenegro belong to the same cluster). Red triangles represent pairs of populations within Eastern Serbia, and blue triangles pairs of populations from Eastern Serbia with Montenegrin population. (TIF) [file pone.0210321.s006.tif]
