# Supplementary material for: Population genetic structure of the Mediterranean horseshoe bat Rhinolophus euryale in the central Balkans
Source: PLoS One. 2019 Jan 30;14(1):e0210321. doi: 10.1371/journal.pone.0210321 (PMC6353099; doi:10.1371/journal.pone.0210321)
Supplement: S1 Table — (DOC) [file pone.0210321.s007.doc]

|  | 1 | 2 | 3 | 4 | 5 | 6 | 7 | 8 | 9 | 10 | 11 | 12 |
| --- | --- | --- | --- | --- | --- | --- | --- | --- | --- | --- | --- | --- |
| 1 |  | 0.344 | 0.242 | **0.026** | 0.653 | 0.562 | 0.202 | **0.000** | **0.000** | **0.008** | 0.932 | **0.000** |
| 2 | 0.004 |  | 0.423 | 0.611 | 0.642 | 0.698 | 0.443 | **0.003** | **0.040** | 0.143 | 0.157 | **0.000** |
| 3 | 0.004 | 0.022 |  | 0.449 | 0.343 | 0.688 | 0.368 | **0.000** | 0.063 | 0.108 | 0.140 | **0.000** |
| 4 | **0.015** | -0.001 | 0.001 |  | **0.043** | 0.679 | 0.164 | **0.001** | **0.015** | 0.088 | 0.284 | **0.000** |
| 5 | -0.002 | -0.001 | 0.003 | **0.015** |  | 0.472 | 0.388 | **0.002** | **0.014** | 0.074 | 0.299 | **0.000** |
| 6 | 0.000 | -0.002 | -0.002 | -0.002 | 0.002 |  | 0.860 | **0.002** | **0.001** | **0.044** | 0.624 | **0.000** |
| 7 | 0.005 | 0.002 | 0.002 | 0.005 | 0.003 | -0.004 |  | **0.000** | **0.012** | 0.077 | 0.116 | **0.000** |
| 8 | **0.036** | **0.027** | **0.024** | **0.024** | **0.032** | **0.024** | **0.020** |  | 0.595 | 0.069 | **0.016** | **0.000** |
| 9 | **0.041** | **0.019** | 0.012 | **0.022** | **0.026** | **0.029** | **0.021** | 0.000 |  | 0.393 | **0.009** | **0.000** |
| 10 | **0.042** | 0.016 | 0.014 | 0.019 | 0.024 | **0.023** | 0.018 | 0.020 | 0.007 |  | **0.003** | **0.000** |
| 11 | -0.011 | 0.011 | 0.010 | 0.006 | 0.008 | -0.001 | 0.011 | **0.026** | **0.032** | **0.058** |  | **0.000** |
| 12 | **0.055** | **0.052** | **0.045** | **0.045** | **0.050** | **0.052** | **0.058** | **0.054** | **0.062** | **0.064** | **0.054** |  |

Values in bold indicate differentiations that are significantly greater than expected by random at p < 0.05
