# Supplementary material for: Population genetic structure of the Mediterranean horseshoe bat Rhinolophus euryale in the central Balkans
Source: PLoS One. 2019 Jan 30;14(1):e0210321. doi: 10.1371/journal.pone.0210321 (PMC6353099; doi:10.1371/journal.pone.0210321)
Supplement: S2 Table — (DOC) [file pone.0210321.s008.doc]

|  | 1 | 2 | 3 | 4 | 5 | 6 | 7 | 8 | 9 | 10 | 11 | 12 |
| --- | --- | --- | --- | --- | --- | --- | --- | --- | --- | --- | --- | --- |
| 1 |  | 0.337 | 0.240 | **0.029** | 0.667 | 0.543 | 0.199 | **0.002** | **0.001** | **0.002** | 0.957 | **0.001** |
| 2 | 0.009 |  | 0.442 | 0.639 | 0.664 | 0.689 | 0.389 | **0.003** | **0.045** | 0.123 | 0.162 | **0.003** |
| 3 | 0.014 | 0.001 |  | 0.470 | 0.354 | 0.672 | 0.404 | **0.002** | 0.072 | 0.067 | 0.161 | **0.002** |
| 4 | **0.057** | -0.012 | 0.000 |  | **0.035** | 0.728 | 0.180 | **0.001** | **0.015** | 0.068 | 0.279 | **0.005** |
| 5 | -0.014 | -0.017 | 0.006 | **0.052** |  | 0.499 | 0.385 | **0.003** | **0.012** | 0.051 | 0.302 | **0.007** |
| 6 | -0.007 | -0.015 | -0.011 | -0.015 | -0.001 |  | 0.853 | **0.003** | **0.003** | **0.029** | 0.614 | **0.001** |
| 7 | 0.019 | 0.003 | 0.002 | 0.016 | 0.004 | -0.019 |  | **0.006** | **0.011** | **0.033** | 0.116 | **0.001** |
| 8 | **0.132** | **0.098** | **0.090** | **0.095** | **0.112** | **0.091** | **0.068** |  | 0.517 | **0.049** | **0.018** | **0.002** |
| 9 | **0.153** | **0.060** | 0.040 | **0.076** | **0.087** | **0.103** | **0.066** | -0.006 |  | 0.334 | **0.010** | **0.001** |
| 10 | **0.163** | 0.051 | 0.062 | 0.075 | 0.084 | **0.096** | **0.070** | **0.077** | 0.013 |  | **0.005** | **0.004** |
| 11 | -0.052 | 0.037 | 0.033 | 0.019 | 0.018 | -0.014 | 0.036 | **0.091** | **0.112** | **0.214** |  | **0.011** |
| 12 | **0.151** | **0.127** | **0.112** | **0.106** | **0.125** | **0.128** | **0.162** | **0.144** | **0.175** | **0.199** | **0.143** |  |

Values in bold indicate differentiations that are significantly greater than expected by random at p < 0.05
