# Supplementary material for: Population genetic structure of the Mediterranean horseshoe bat Rhinolophus euryale in the central Balkans
Source: PLoS One. 2019 Jan 30;14(1):e0210321. doi: 10.1371/journal.pone.0210321 (PMC6353099; doi:10.1371/journal.pone.0210321)
Supplement: S3 Table — (DOC) [file pone.0210321.s009.doc]

|  | 1 | 2 | 3 | 4 | 5 | 6 | 7 | 8 | 9 | 10 | 11 | 12 |
| --- | --- | --- | --- | --- | --- | --- | --- | --- | --- | --- | --- | --- |
|  |  | 0.336 | 0.241 | **0.029** | 0.667 | 0.543 | 0.199 | **0.002** | **0.001** | **0.001** | 0.957 | **0.001** |
| 2 | 0.007 |  | 0.442 | 0.639 | 0.664 | 0.689 | 0.389 | **0.003** | **0.045** | 0.122 | 0.162 | **0.002** |
| 3 | 0.010 | 0.001 |  | 0.470 | 0.354 | 0.672 | 0.404 | **0.002** | **0.075** | 0.069 | 0.161 | **0.002** |
| 4 | 0.044 | -0.009 | 0.000 |  | **0.036** | 0.728 | 0.179 | **0.001** | **0.016** | 0.068 | 0.278 | **0.005** |
| 5 | -0.011 | -0.012 | 0.004 | **0.040** |  | 0.499 | 0.385 | **0.003** | **0.012** | 0.052 | 0.302 | **0.007** |
| 6 | -0.005 | -0.011 | -0.008 | -0.011 | 0.000 |  | 0.854 | **0.003** | **0.003** | **0.030** | 0.615 | **0.001** |
| 7 | 0.014 | 0.002 | 0.001 | 0.012 | 0.003 | -0.015 |  | **0.006** | **0.011** | **0.031** | 0.115 | **0.001** |
| 8 | **0.102** | **0.074** | **0.069** | **0.073** | **0.086** | **0.070** | **0.052** |  | 0.517 | **0.049** | **0.018** | **0.002** |
| 9 | **0.119** | **0.045** | **0.030** | **0.058** | **0.066** | **0.079** | **0.050** | -0.005 |  | 0.334 | **0.010** | **0.001** |
| 10 | **0.126** | 0.038 | 0.047 | 0.057 | 0.063 | **0.073** | **0.052** | **0.058** | 0.010 |  | **0.005** | **0.004** |
| 11 | -0.039 | 0.028 | 0.025 | 0.014 | 0.014 | -0.011 | 0.028 | **0.070** | **0.086** | **0.168** |  | **0.011** |
| 12 | **0.119** | **0.099** | **0.088** | **0.083** | **0.097** | **0.100** | **0.127** | **0.113** | **0.137** | **0.156** | **0.113** |  |

Values in bold indicate differentiations that are significantly greater than expected by random at p < 0.05
