# Supplementary material for: Population genetic structure of the Mediterranean horseshoe bat Rhinolophus euryale in the central Balkans
Source: PLoS One. 2019 Jan 30;14(1):e0210321. doi: 10.1371/journal.pone.0210321 (PMC6353099; doi:10.1371/journal.pone.0210321)
Supplement: S4 Table — (DOC) [file pone.0210321.s010.doc]

|  | 1 | 2 | 3 |
| --- | --- | --- | --- |
| 1 |  | **0.112** | **0.268** |
| 2 | **0.130** |  | **0.253** |
| 3 | **0.304** | **0.286** |  |

1 – Eastern Serbia and Montenegro; 2 – Western Serbia; 3 – Slovenia. Values in bold indicate differentiations that are significantly greater than expected by random at p < 0.001
